# New records of anisakid nematodes from marine fishes off New Caledonia, with descriptions of five new species of *Raphidascaris* (*Ichthyascaris*) (Nematoda, Anisakidae)

**DOI:** 10.1051/parasite/2020016

**Published:** 2020-03-30

**Authors:** František Moravec, Jean-Lou Justine

**Affiliations:** 1 Institute of Parasitology, Biology Centre of the Czech Academy of Sciences Branišovská 31 370 05 České Budějovice Czech Republic; 2 Institut Systématique, Évolution, Biodiversité (ISYEB), Muséum National d’Histoire Naturelle, CNRS, Sorbonne Université, EPHE, Université des Antilles Rue Cuvier, CP 51 75005 Paris France

**Keywords:** Nematode parasite, Ascaridoidea, Aulopiformes, Elopiformes, Perciformes, South Pacific

## Abstract

Recent examinations of anisakid nematodes (Anisakidae) from marine fishes off New Caledonia, collected in the years 2003–2008, revealed the presence of the following five new species of *Raphidascaris* Railliet et Henry, 1915, all belonging to the subgenus *Ichthyascaris* Wu, 1949: *Raphidascaris* (*Ichthyascaris*) *spinicauda* n. sp. from the redbelly yellowtail fusilier *Caesio cuning* (Caesionidae, Perciformes); *Raphidascaris* (*Ichthyascaris*) *fasciati* n. sp. from the blacktip grouper *Epinephelus fasciatus* (Serranidae, Perciformes); *Raphidascaris* (*Ichthyascaris*) *nudicauda* n. sp. from the brushtooth lizardfish *Saurida undosquamis* (Synodontidae, Aulopiformes); *Raphidascaris* (*Ichthyascaris*) *euani* n. sp. from the Japanese large-eye bream *Gymnocranius euanus* (Lethrinidae, Perciformes); and *Raphidascaris* (*Ichthyascaris*) *elopsis* n. sp. from the Hawaiian ladyfish *Elops hawaiensis* (Elopidae, Elopiformes). An additional two congeneric species, *R*. (*I*.) *etelidis* Moravec et Justine, 2012 and *R*. (*I*.) *sillagoides* (Bruce, 1990) were found in the deep-water red snapper *Etelis carbunculus* (new host record) and the deepwater longtail red snapper *Etelis coruscans* (both Lutjanidae, Perciformes), and the silver sillago *Sillago sihama* (Sillaginidae, Perciformes) (new host and geographical records), respectively. Two unidentified congeneric species, *Raphidascaris* (*Ichthyascaris*) sp. 1 from the trumpet emperor *Lethrinus miniatus* (Lethrinidae, Perciformes) and *Raphidascaris* (*Ichthyascaris*) sp. 2 from the white-spotted puffer *Arothron hispidus* (Tetraodontidae, Tetraodontiformes) were recorded. Moreover, two species of *Hysterothylacium* Ward et Magath, 1917, *H*. *alatum* Moravec et Justine, 2015 and *H*. *epinepheli* (Yamaguti, 1941), were found in the leopard coralgrouper *Plectropomus leopardus* (type host) and the highfin grouper *Epinephelus maculatus* (new host) (both Serranidae, Perciformes), respectively. This is the second finding of *H*. *epinepheli* since its original description in Japan 79 years ago. Most species are described based on light and electron microscopical studies.

## Abstract (fr)

L’examen récent de nématodes Anisakidae de poissons marins de la Nouvelle-Calédonie, collectés dans les années 2003–2008, a révélé la présence des cinq nouvelles espèces de *Raphidascaris* Railliet et Henry, 1915, toutes appartenant au sous-genre *Ichthyascaris* Wu, 1949 : *Raphidascaris (Ichthyascaris) spinicauda* n. sp. chez le fusilier *Caesio cuning* (Caesionidae, Perciformes) ; *Raphidascaris (Ichthyascaris) fasciati* n. sp. chez la loche *Epinephelus fasciatus* (Serranidae, Perciformes) ; *Raphidascaris (Ichthyascaris) nudicauda* n. sp. chez le poisson-lézard *Saurida undosquamis* (Synodontidae, Aulopiformes) ; *Raphidascaris (Ichthyascaris) euani* n. sp. chez le bossu *Gymnocranius euanus* (Lethrinidae, Perciformes) ; et *Raphidascaris (Ichthyascaris) elopsis* n. sp. chez *Elops hawaiensis* (Elopidae, Elopiformes). Deux autres espèces congénériques, *R. (I.) etelidis* Moravec et Justine, 2012 et *R. (I.) sillagoides* (Bruce, 1990) ont été trouvées respectivement chez les vivaneaux de profondeur *Etelis carbunculus* (nouvel hôte) et *Etelis coruscans* (Lutjanidae, Perciformes) et chez *Sillago sihama* (Sillaginidae, Perciformes) (nouvel hôte et nouvelle mention géographique). Deux espèces congénériques non identifiées, *Raphidascaris (Ichthyascaris)* sp. 1 chez le bossu *Lethrinus miniatus* (Lethrinidae, Perciformes) et *Raphidascaris (Ichthyascaris)* sp. 2 chez *Arothron hispidus* (Tetraodontidae, Tetraodontiformes) sont signalées. De plus, deux espèces d’*Hysterothylacium* Ward et Magath, 1917, *H. alatum* Moravec et Justine, 2015 et *H. epinepheli* (Yamaguti, 1941), ont été trouvées chez la saumonée *Plectropomus leopardus* (hôte-type) et chez la loche uitoé *Epinephelus maculatus* (nouvel hôte) (Serranidae, Perciformes), respectivement. Il s’agit de la deuxième mention d’*H. epinepheli* depuis sa description originale au Japon il y a 79 ans. La plupart des espèces ont été décrites sur la base d’études au microscope optique et électronique.

## Introduction

To date, only six nominal species of adult anisakid nematodes (Anisakidae) have been reported from marine teleost fishes and elasmobranchs in New Caledonian waters: *Hysterothylacium alatum* Moravec et Justine, 2015, *H*. *cenaticum* (Bruce et Cannon, 1989), *H*. *sphyraenae* Moravec et Justine, 2015, *Raphidascaris* (*Ichthyascaris*) *etelidis* Moravec et Justine, 2012, *R*. (*I*.) *nemipteri* 2005, and *Terranova scoliodontis* (Baylis, 1931) [9, 17–19, 21]. Moreover, an additional adult anisakid nematode not identified to species, reported as *Raphidascaris* (*Ichthyascaris*) sp., was recorded from *Carangoides dinema* Bleeker and *C*. *fulvoguttatus* (Forsskål) (Carangidae) [23]. Anisakid nematodes unidentified to species or genera, mostly as larval stages, have also been reported from fishes belonging to different families by Justine et al. [10–12] and Shamsi et al. [25–27].

Recent examinations of adult anisakid nematodes collected by J.-L. Justine and his students in marine fishes from off New Caledonia in the years 2003–2008 revealed the presence of five previously unknown and one known species of *Raphidascaris* Railliet et Henry, 1915 (subgenus *Ichthyascaris* Wu, 1949), the latter representing new host and geographical records, and two known species of *Hysterothylacium* Ward et Magath, 1917, the finding of one of which also represents new host and geographical records. Results of this study are presented herein.

## Materials and methods

Fish were caught off New Caledonia by various means; those obtained from the fishmarket in Nouméa were very fresh and thus were probably fished in the near vicinity. The nematodes were generally collected with the “wash” method and were fixed in hot 4% formalin or 70% ethanol [13]. For light microscopical (LM) examination, they were cleared with glycerine. Drawings were made with the aid of a Zeiss microscope drawing attachment. Specimens used for scanning electron microscopical (SEM) examination were postfixed in 1% osmium tetroxide (in phosphate buffer), dehydrated through a graded acetone series, critical-point-dried and sputter-coated with gold; they were examined using a JEOL JSM-7401F scanning electron microscope at an accelerating voltage of 4 kV (GB low mode). All measurements are in micrometres unless otherwise indicated. The classification system of the Ascaridoidea adopted follows Keys to the Nematode Parasites of Vertebrates [1, 7]. The fish nomenclature follows FishBase [6].

## Results

Family Anisakidae Railliet et Henry, 1912

### *Raphidascaris* (*Ichthyascaris*) *spinicauda* n. sp. Figures 1 and 2

urn:lsid:zoobank.org:act:88EFDC0B-768A-4F0F-8D07-37E9A5307EBB

**Figure 1:**
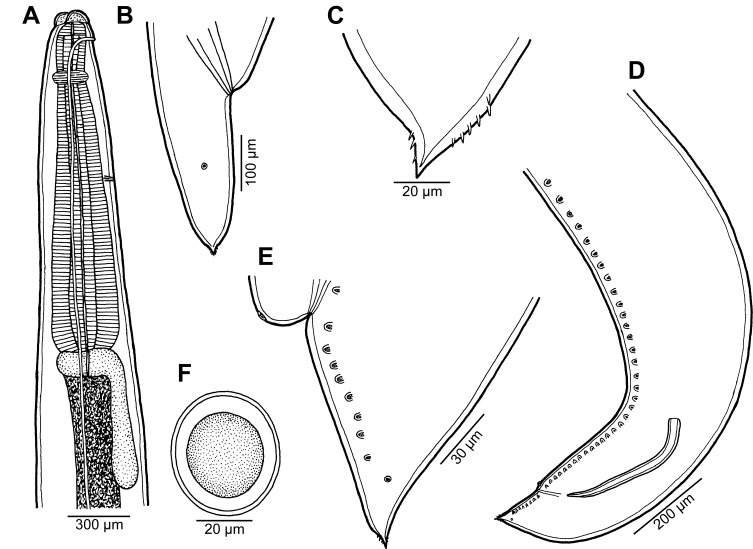
*Raphidascaris* (*Ichthyascaris*) *spinicauda* n. sp. ex *Caesio cuning*. (A) Anterior end of female, lateral view; (B) tail of female, lateral view; (C) tail tip of female, lateral view; (D) posterior end of male, lateral view; (E) tail of male, lateral view; (F) egg.

**Figure 2:**
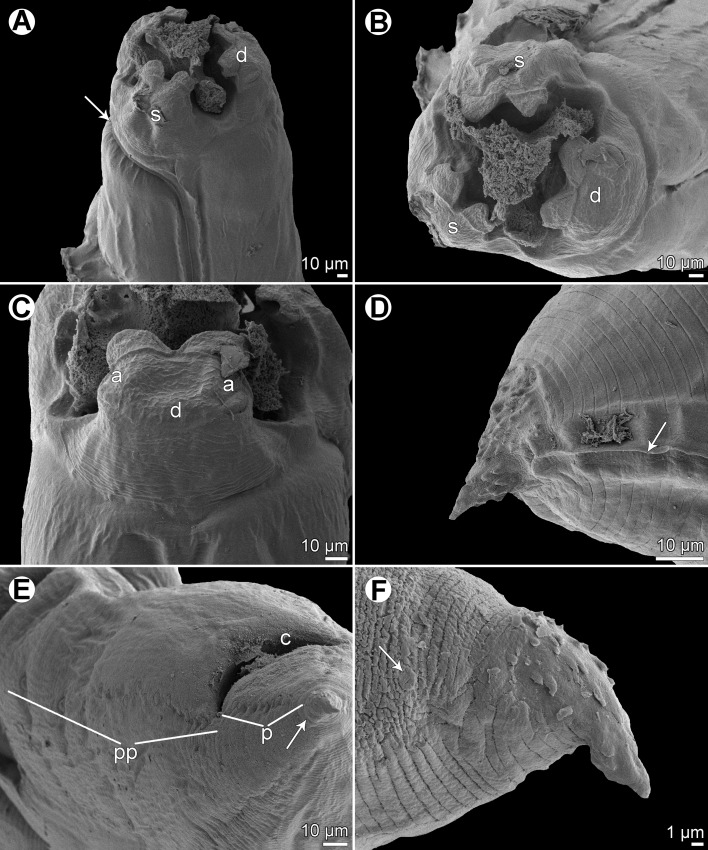
*Raphidascaris* (*Ichthyascaris*) *spinicauda* n. sp., scanning electron micrographs. (A) Cephalic end of female, sublateral view (arrow indicates ventral connection of lateral alae); (B) cephalic end of female, apical view; (C) dorsal lip, dorsal view; (D) tail tip of female, lateral view (upper side is ventral) (arrow indicates lateral ala); (E) posterior end of male, ventrolateral view (arrow indicates phasmid); (F) tail tip of male, lateral view (upper side is ventral) (arrow indicates phasmid). (a) Double cephalic papilla; (c) cloaca; (d) dorsal lip; (p) postanal papillae; (pp) preanal papillae; (s) subventral lip.

Type host: Redbelly yellowtail fusilier *Caesio cuning* (Bloch) (Caesionidae, Perciformes).

Site of infection: Intestine.

Type locality: Fish market, Nouméa, New Caledonia (collected 16 December 2008).

Prevalence, intensity and details about fish: 1 fish infected/5 fish examined the same day; 2 nematodes. The infected fish, JNC2853, was 220 mm in fork length and 216 g in weight; its photograph has been deposited in Wikimedia (https://commons.wikimedia.org/wiki/File:Caesio_cuning.jpg).

Etymology: The specific name *spinicauda* is the Latin noun in apposition, composed of two words, *spina* (= spine) and *cauda* (= tail), and relates to the characteristic feature of this species, i.e. the presence of cuticular spines on the tail tip.

Deposition of type specimens: Holotype and allotype mounted on SEM stub in the Helminthological Collection, Institute of Parasitology, Biology Centre of the Czech Academy of Sciences, České Budějovice, Czech Republic (Cat. No. N–1214).

#### Description

*General*: Medium-sized nematodes with transversely striated cuticle (Figs. 2D and 2F). Lips nearly equal in size, without lateral membranous flanges; pulp with 2 distinct anterior lobes, each with terminal pocket-like depression (Figs. 2A–2C). Dorsal lip bears 2 subdorsal double papillae (Fig. 2C); each ventrolateral lip with 1 double subventral papilla, 1 small single papilla and amphid situated laterally. Interlabia absent. Narrow lateral alae extend along whole body length, united anteriorly close to ventrolateral lips on 1 side of body (Figs. 1A and 2A). Oesophagus short; posterior half markedly broad in female (Fig. 1A). Ventriculus transversely oval; ventricular appendix relatively short (Fig. 1A). Excretory pore well posterior to level of nerve ring (Fig. 1A). Tail of both sexes conical.

*Male* (1 specimen without cephalic end, holotype): Length of incomplete body 10.17 mm, maximum width 544. Length of incomplete oesophagus 748, maximum width 218. Ventriculus 109 × 163; ventricular appendix 394 long, 82 in maximum width. Posterior end curved ventrally. Spicules equal, alate, pointed, 309 long, representing probably around 3% of body length. Total of 43 pairs of small subventral papillae present, 35 being preanals and 8 postanals; papillae of approximately 13 posteriormost preanal pairs and of postanal pairs very small; postanal papillae of third pair from posterior extremity single (not doubled) (Figs. 1D, 1E and 2E). Anterior cloacal lip with poorly developed unpaired median papilla. Pair of small lateral phasmids present, located anterior to tail tip (Figs. 1D, 1E, 2E and 2F). Tail 120 long, its tip provided with many cuticular spines or protuberances, mostly on its ventral side (Fig. 2F).

*Female* (1 ovigerous specimen, allotype): Length of body 16.37 mm, maximum width 639. Lips 78 long. Length of oesophagus 1.59 mm, representing 9.7% of body length, maximum width 326. Nerve ring and excretory pore 340 and 816, respectively, from anterior extremity. Ventriculus 122 × 286; ventricular appendix 625 long, maximum width 136. Vulva situated in anterior region of body, 3.43 mm from anterior extremity, at 21% of body length; vagina directed posteriorly from vulva. Uterus forms coils in region posterior to vagina, extending posteriorly to level of rectum. Eggs numerous, suboval to almost rounded, thin-walled, with uncleaved contents (Fig. 1F); size 45–54 × 42–48. Tail 408 long; tip with numerous minute cuticular spines distributed mainly on ventral side (Figs. 1C and 2D).

#### Remarks

At present, there are eleven species in the subgenus *Ichthyascaris* Wu, 1949 of *Raphidascaris* Railliet et Henry, 1915 in the conception of Moravec and Nagasawa [22]: *R*. *arii* Yooyen, Moravec et Wongsawad, 2011, *R*. *chirocentri* Yamaguti, 1935, *R*. *etelidis* Moravec et Justine, 2012, *R*. *fisheri* (Hooper, 1983), *R*. *gymnocraniae* (Bruce, 1990), *R*. *longispicula* Li, Liu, Liu et Zhang, 2012, *R*. *lophii* (Wu, 1949), *R*. *nemipteri* Moravec et Justine, 2005, *R*. *sillagoides* (Bruce, 1990), *R*. *trichiuri* (Yin et Zhang, 1983) and *R*. *vicentei* Santos, 1970 [14, 19]. Li et al. [14] also listed *R*. *lutiani* Olsen, 1952 (misspelled as *lutjani*) in this subgenus, but the main morphological feature of *Ichthyascaris* (i.e., anteriorly united lateral alae) [22] is not apparent from the original description of this North American species [24], so that it is not included in this subgenus.

Sheenko [28] designated *R*. *lophii* and *R*. *trichiuri* as the junior synonyms of *R*. *chirocentri*, but this conclusion was based solely on available inadequate species descriptions. Therefore, both these species are, for the time being, dealt with as valid in the present paper. The two males and one female of *Raphidascaris* (*Ichthyascaris*), collected from the intestine of *Etelis carbunculus* Cuvier (reported as *E*. *marshi*) (Lutjanidae) off the Philippines, were identified and briefly described by Sheenko [28] as *Ichthyascaris* [= *R*. (*I*.)] *chirocentri*, but this identification seems to be questionable.

The new species, *R*. (*I*.) *spinicauda* n. sp., differs distinctly from *R*. *fisheri* and *R*. *trichiuri* in the presence of numerous small cuticular spines on the female tail tip; from the former species also by the absence of a small bulge posterior to the anterolateral sockets on the lateral margins of the lips [5, 16]. From *R*. *lophii* and *R*. *longispicula* it differs in much shorter spicules (309 μm vs. 540–690 μm and 1.13–1.32 mm, respectively) [14, 30], from *R*. *vicentei* it can be differentiated by the male tail tip with spines (vs. aspinose), slightly longer spicules (309 μm vs. 125–300 μm), less numerous pairs of postanal papillae (8 pairs vs. 10–11 pairs) and by less elongate lips with protruding inner lobes (vs. more elongate lips without markedly protruding inner lobes), whereas from *R*. *chirocentri* by less numerous pairs of all caudal papillae and those of postanal papillae (43 and 8 vs. 63 and 13) [29, 31].

In contrast to the new species, the male tail tip of *R*. *nemipteri* is smooth (vs. spinose) and the postanal papillae of the third pair from the posterior extremity are doubled (vs. single) [17]. The male tail tip of *R*. *arii*, *R*. *etelidis*, *R*. *gymnocraniae*, and *R*. *sillagoides* is spinose as in the new species, but the postanal papillae of the third pair from the posterior extremity in *R*. *gymnocraniae* and *R*. *sillagoides* are single (vs. doubled), and they have fewer pairs of all caudal papillae (33–38 and 31–37, respectively, vs. 43) [5]. The postanal papillae of the third pair from the posterior extremity in *R*. *arii* and *R*. *etelidis* are doubled (vs. single); moreover, the former species differs in fewer pairs of preanal papillae (21–30 vs. 43) and the latter one in longer spicules (345–474 μm vs. 309 μm), and more pairs of preanal (44–49 vs. 35) and postanal (12–13 vs. 8) papillae [19, 33]. *Raphidascaris spinicauda* n. sp. is the first species of the subgenus *Ichthyascaris* reported from a fish host belonging to the family Caesionidae.

Previous records of *Raphidascaris* (*Ichthyascaris*) spp. from fishes in New Caledonian waters include *R*. *nemipteri* from *Nemipterus furcosus* (Valenciennes) (Nemipteridae), *R*. *etelidis* from *Etelis coruscans* Valenciennes and *Pristipomoides filamentosus* (Valenciennes) (both Lutjanidae), and *Raphidascaris* (*I*.) sp. from *Carangoides dinema* Bleeker and *C*. *fulvoguttatus* (Forsskål) (both Carangidae) [17, 19, 23].

### *Raphidascaris* (*Ichthyascaris*) *fasciati* n. sp. Figures 3–5

urn:lsid:zoobank.org:act:082F05ED-D367-499A-A5C6-4C015310BB43

**Figure 3:**
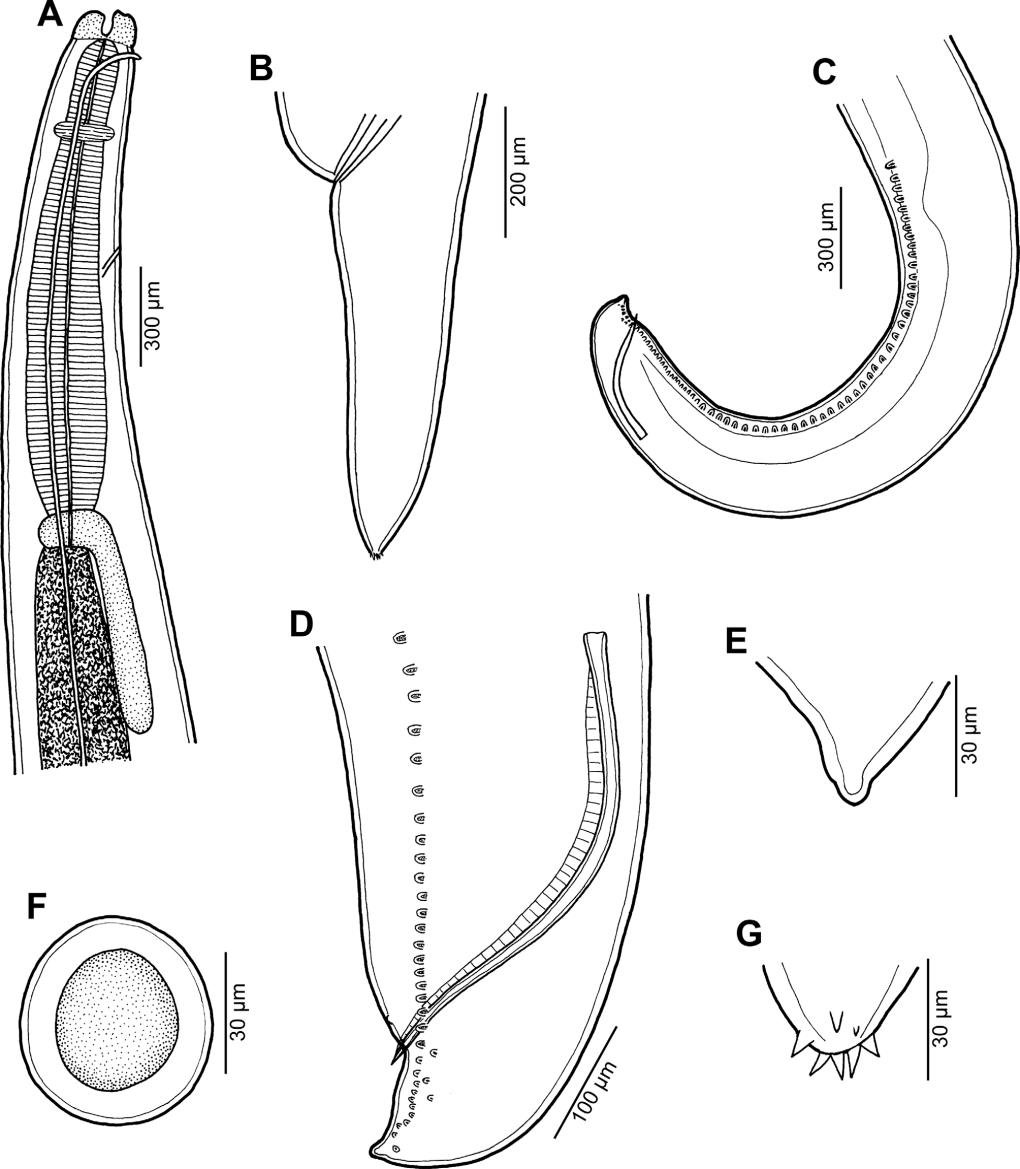
*Raphidascaris* (*Ichthyascaris*) *fasciati* n. sp. ex *Epinephelus fasciatus*. (A) Anterior end of male, lateral view; (B) tail of gravid female, lateral view; (C) posterior end of male, lateral view; (D) caudal end of male, lateral view; (E) tail tip of male; (F) egg; (G) tail tip of female.

Type host: Blacktip grouper *Epinephelus fasciatus* (Forsskål) (Serranidae, Perciformes).

Site of infection: Intestine.

Type locality: External slope of Récif Kué, off New Caledonia, 22°34′702 S, 166°29′072 E (collected 22 August 2007).

Prevalence, intensity and details about fish: 1 fish infected/61 fish examined [12]; 92 nematodes. The infected fish, JNC2280, was 280 mm in fork length and 326 g in weight.

Etymology: The specific name of this nematode relates to the genitive form of the species name of the type host.

Deposition of type specimens: Muséum National d’Histoire Naturelle, Paris, France (holotype, allotype and 85 paratypes, MNHN JNC2280A, JNC2280B) and Helminthological Collection, Institute of Parasitology, Biology Centre of the Czech Academy of Sciences, České Budějovice, Czech Republic (2 paratypes, N–1215).

#### Description

**Figure 4:**
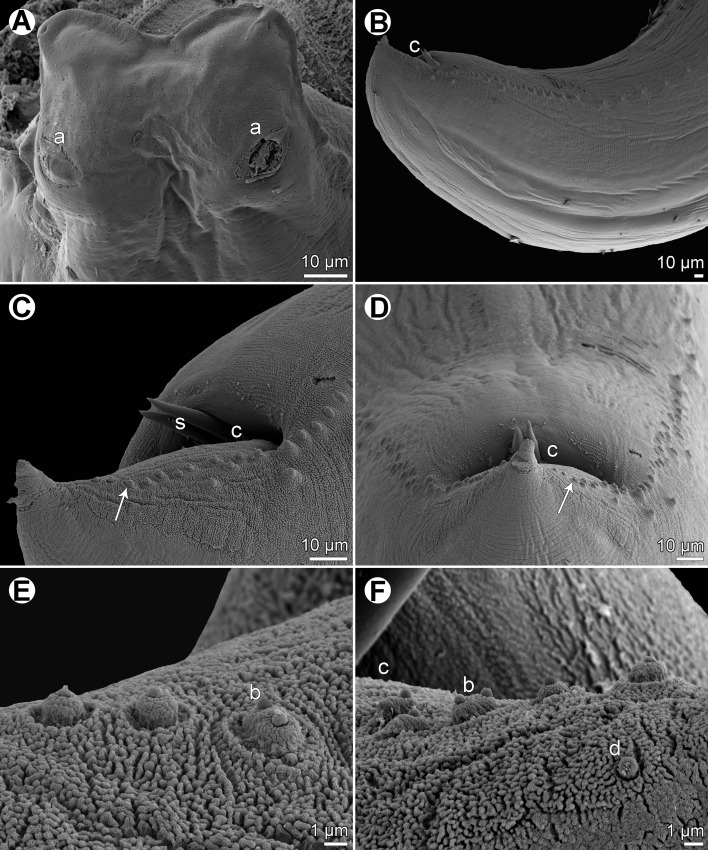
*Raphidascaris* (*Ichthyascaris*) *fasciati* n. sp., scanning electron micrographs of male. (A) Dorsal lip; (B) posterior end of male, lateral view; (C) tail, sublateral view (arrow indicates postanal double papilla); (D) region of cloaca and tail, dorsoventral view (arrow indicates postanal double papilla); (E) postanal papillae of three posteriormost pairs; (F) region of postanal papillae of four posteriormost pairs, lateral view. (a) Labial double papilla; (b) postanal double papilla; (c) cloacal aperture; (d) phasmid; (s) spicules.

**Figure 5:**
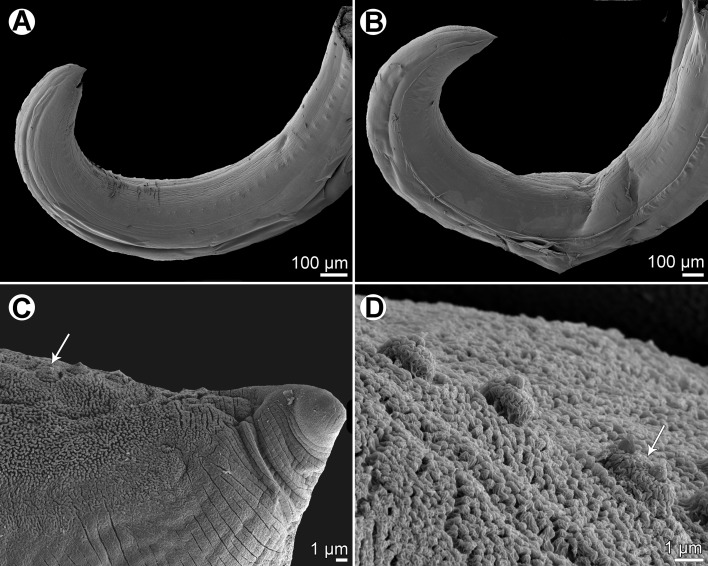
*Raphidascaris* (*Ichthyascaris*) *fasciati* n. sp., scanning electron micrographs of male. (A and B) Posterior end of body, lateral views (two different specimens); (C) posterior end of tail, lateral view (arrow indicates postanal double papilla) (note different structure of cuticle on ventral and dorsal sides); (D) postanal papillae of three posteriormost pairs (arrow indicates double papilla).

*General*: Medium-sized nematodes with transversely striated cuticle (Figs. 4C, 4D and 5C). Lips approximately equal in size, without lateral membranous flanges; pulp with 2 moderately developed anterior lobes, each with terminal pocket-like depression. Dorsal lip bears 2 subdorsal double papillae (Fig. 4A); each ventrolateral lip with 1 double subventral papilla, 1 small single papilla and amphid situated laterally. Interlabia absent. Narrow lateral alae extend along whole body length, united anteriorly close to ventrolateral lips on 1 side of body (Fig. 3A). Oesophagus short; posterior half markedly broad (Fig. 3A). Ventriculus transversely oval; ventricular appendix relatively short (Fig. 3A). Excretory pore well posterior to level of nerve ring (Fig. 3A). Tail of both sexes conical.

*Male* (5 specimens; measurements of holotype in parentheses): Length of body 18.09–20.66 (20.06) mm, maximum width 571–748 (680). Lips 109–122 (109) long. Length of oesophagus 1.61–1.88 (1.63) mm, maximum width 245–326 (286). Nerve ring and excretory pore 462–544 (503) and 639–816 (694), respectively, from anterior extremity. Ventriculus 122–150 × 177–245 (150 × 245); ventricular appendix 381–721 (721) long, 82–109 (109) in maximum width. Posterior end curved ventrally. Spicules equal, alate, pointed, 420–543 (543) long, representing 2.2–2.9 (2.7)% of body length. Total of 73–75 (75) pairs of subventral papillae present, 59–61 (60) being preanal, 1 (1) pair adanal and 13 (13) pairs postanal; papillae of 23–24 (24) posteriormost preanal pairs and of postanal pairs very small, of which approximately 2 pairs of preanals situated more laterally than others; postanal papillae of third pair from posterior extremity doubled (Figs. 3C, 3D, 4C–4F, 5C and 5D). Anterior cloacal lip with poorly developed unpaired median papilla. Pair of small lateral phasmids present, located anterior to tail tip (Figs. 3D and 4F). Tail 96–138 (138) long, its tip rounded, smooth, without cuticular spines or protuberances (Figs. 3D, 3E, 4C, 4D and 5C).

*Female* (5 ovigerous specimens; measurements of allotype in parentheses): Length of body 26.04–32.12 (32.12) mm, maximum width 639–1,020 (1,020). Lips 136–177 (177) long. Length of oesophagus 2.18–2.79 (2.58) mm, representing 8–9 (8)% of body length, maximum width 340–517(517). Nerve ring and excretory pore 517–666 (625) and 694–1,197 (1,115), respectively, from anterior extremity. Ventriculus 150–190 × 272–381 (190 × 381); ventricular appendix 639–802 (789) long, maximum width 95–177 (150). Vulva situated in anterior region of body, 4.15–5.58 (5.58) mm from anterior extremity, at 15–17 (17)% of body length; vagina directed posteriorly from vulva. Uterus forms coils in region posterior to vagina, extending posteriorly to level of rectum. Eggs numerous, suboval to almost rounded, thin-walled, with uncleaved contents (Fig. 3F); size 54–63 × 48–54 (54 in diameter). Tail 585–775 (734) long; tip with numerous minute cuticular spines and protuberances distributed mainly on ventral side (Figs. 3B and 3G).

#### Remarks

This new species is characterized by the absence of cuticular outgrowths (spines or protuberances) on the male tail tip (Figs. 3E and 5C). Of the 11 species of *Raphidascaris* (*Ichthyascaris*) (see above), only the following five have the male tail tip smooth, without minute cuticular spines or protuberances: *R*. *chirocentri*, *R*. *fisheri*, *R*. *nemipteri*, *R*. *trichiuri*, and *R*. *vicentei*. However, in contrast to the new species, the female tail tip of *R*. *fisheri* and *R*. *trichiuri* is smooth, without cuticular spines (vs. female tail tip with numerous small spines); moreover, *R*. *fischeri* has lateral margins of the lips with a small bulge posterior to the anterolateral sockets [5] (vs. such structures not present). The remaining three species, *R*. *chirocentri*, *R*. *nemipteri*, and *R*. *vicentei*, have shorter spicules (315 μm, 225–399 μm and 125–300 μm, respectively, vs. 420–543 μm) and also differ in some other features, such as the numbers of genital papillae and body measurements. The type hosts of these five species belong to other fish families than that of the new species (Chirocentridae, Muraenesocidae, Nemipteridae, and Platycephalidae *vs* Serranidae).

*Epinephelus fasciatus* has been thoroughly examined for parasites in New Caledonia with 21 host-parasite combinations reported in 2010 [10], which were subsequently complemented by additional records of nematodes [20], trypanorhynch cestodes [2] and copepods [8]. The description of the present new species exemplifies again the high biodiversity of parasites in coral-reef fish.

### *Raphidascaris* (*Ichthyascaris*) *nudicauda* n. sp. Figures 6–8

urn:lsid:zoobank.org:act:D309DC41-87F0-43A7-8FAB-F5791F835BB6

**Figure 6:**
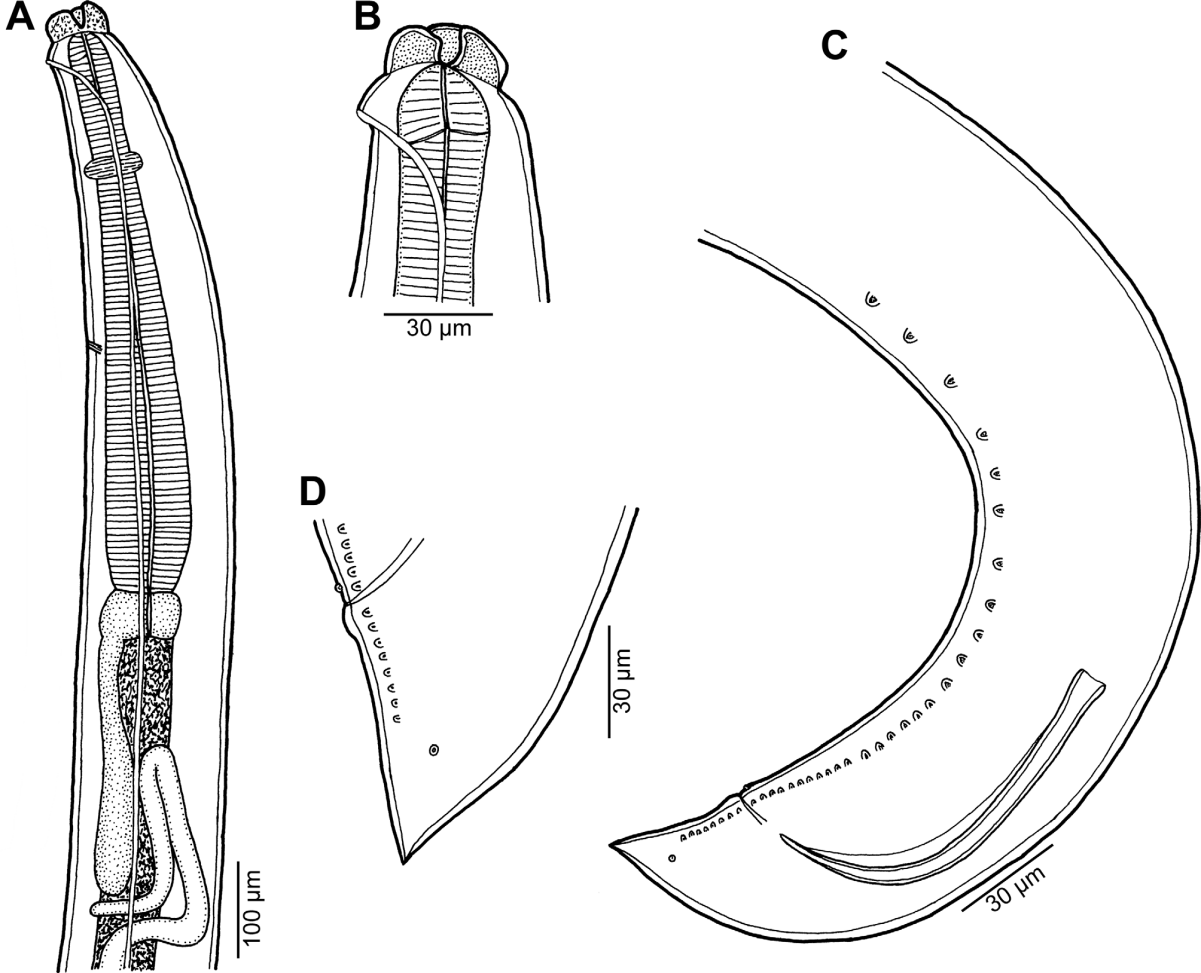
*Raphidascaris* (*Ichthyascaris*) *nudicauda* n. sp. ex *Saurida undosquamis*, male. (A) Anterior end of body, lateral view; (B) cephalic end, lateral view; (C) posterior end of body, lateral view; (D) tail, lateral view.

Type host: Brushtooth lizardfish *Saurida undosquamis* (Richardson) (Synodontidae, Aulopiformes).

Site of infection: Intestine.

Type locality: Baie de Boulari, off Le Mont-Dore, New Caledonia, 22°16′920 S, 166°32′964 E (collected 12 June 2008).

Prevalence, intensity and details about fish: 1 fish infected/6 fish examined the same day; 1 nematode. The infected fish, JNC2591, was 182 mm in length and 52 g in weight.

Etymology: The specific name *nudicauda* is the Latin noun in apposition, composed of two words, *nudus* (= bare, naked) and *cauda* (= tail), and relates to the characteristic feature of this species, i.e. the absence of cuticular spines on the male tail tip.

Deposition of type specimen: Holotype mounted on SEM stub in the Helminthological Collection, Institute of Parasitology, Biology Centre of the Czech Academy of Sciences, České Budějovice, Czech Republic (Cat. No. N–1216).

#### Description

**Figure 7:**
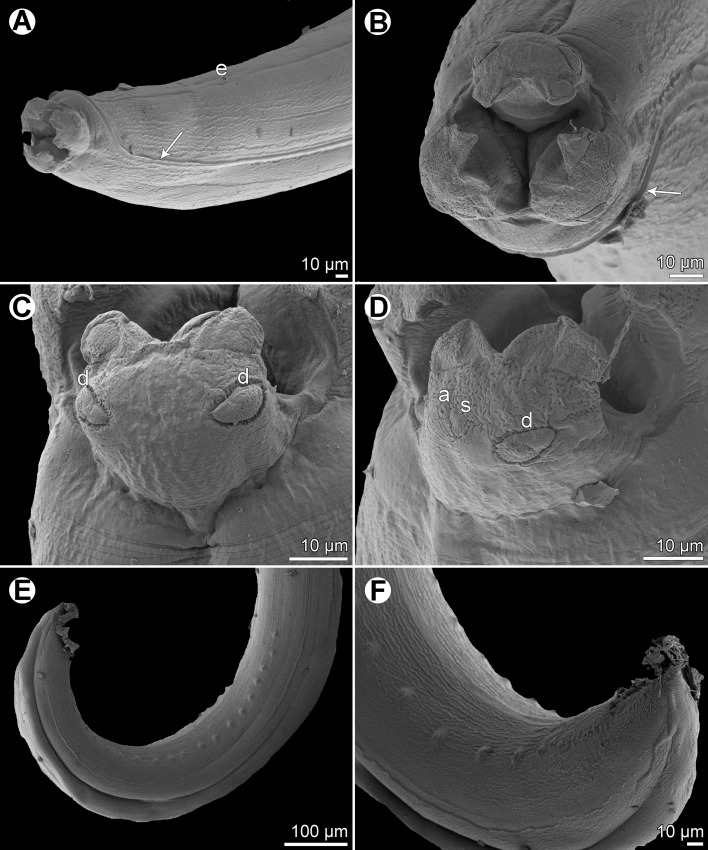
*Raphidascaris* (*Ichthyascaris*) *nudicauda* n. sp., scanning electron micrographs of male. (A) Anterior end of body, sublateral view (arrow indicates lateral ala); (B) cephalic end, apical view (arrow indicates lateral ala); (C) dorsal lip; (D) ventrolateral lip; (E) posterior end, sublateral view; (F) posterior end, ventrolateral view. (a) Amphid; (d) double papilla; (e) excretory pore; (s) single papilla.

**Figure 8:**
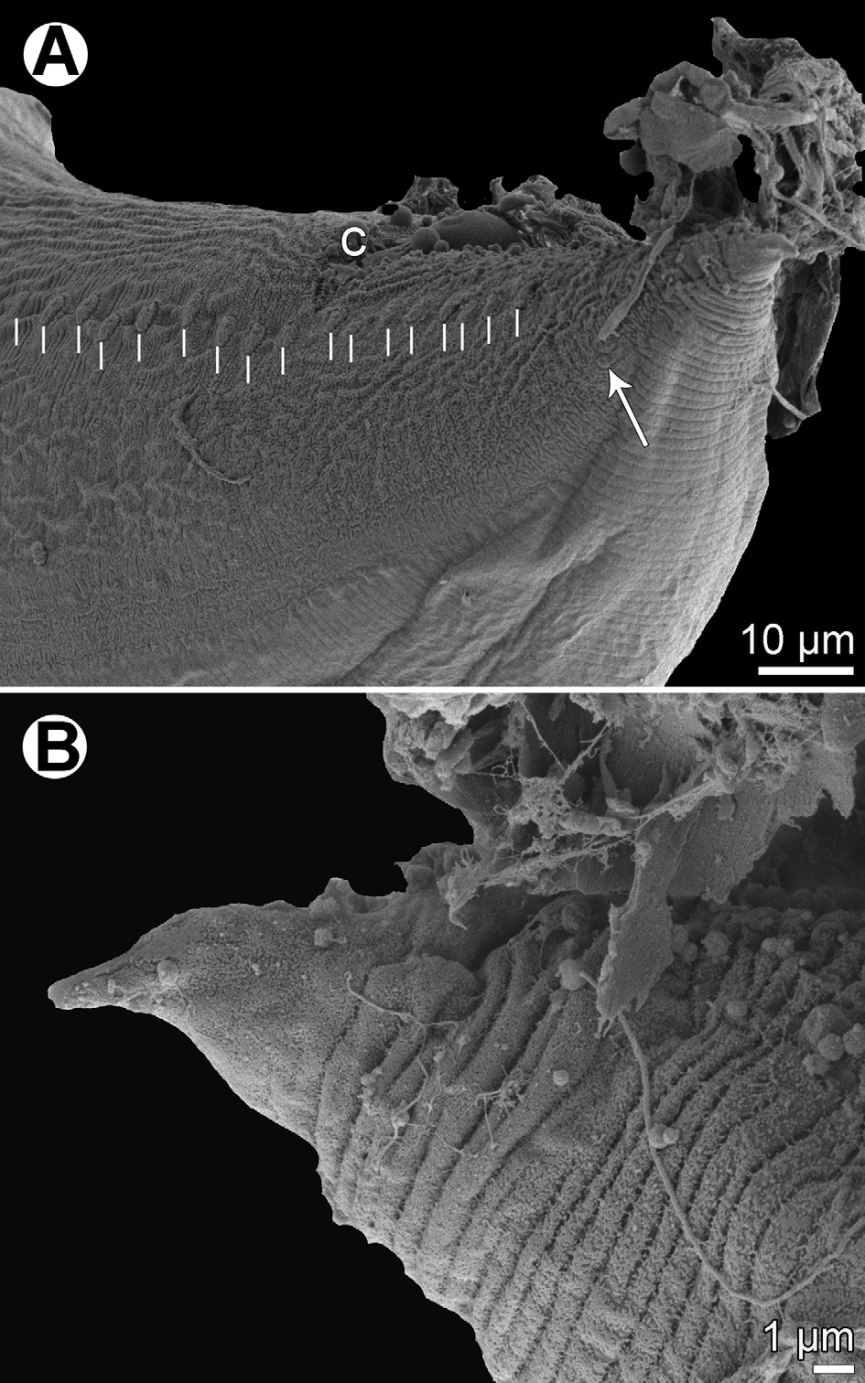
*Raphidascaris* (*Ichthyascaris*) *nudicauda* n. sp., scanning electron micrographs of male. (A) Tail, lateral view (white bars designate caudal papillae; arrow indicates phasmid); (B) tail tip, lateral view.

*Male* (1 specimen, holotype): Small nematode with transversely striated cuticle (Figs. 7C, 8A and 8B). Lips nearly equal in size, without lateral membranous flanges; pulp with 2 distinct anterior lobes, each with terminal pocket-like depression (Figs. 7B–7D). Dorsal lip bears 2 double papillae, each ventrolateral lip with 1 double subventral papilla, 1 small single papilla and amphid situated laterally (Figs. 7B–7D). Interlabia absent. Narrow lateral alae extend along almost whole body length, united anteriorly close to ventrolateral lips on 1 side of body (Figs. 6A, 6B, 7A and 7B). Length of body 5.94 mm, maximum width 150. Oesophagus 680 long, representing 11% of body length, much broader at its posterior half (Fig. 6A). Ventriculus transversely oval, 54 × 95; ventricular appendix 299 long and 41 wide. Nerve ring and excretory pore 204 and 503, respectively, from anterior end of body (Figs. 6A, 7A). Posterior end of body curved ventrally (Figs. 6C, 7E and 7F). Spicules equal, alate, pointed, 201 long, representing 3.4% of body length. Total of 36 pairs of subventral papillae present, 28 being preanals and 8 postanals; papillae of 11 posteriormost preanal pairs and postanal pairs very small; no doubled postanal papillae present (Figs. 6C, 6D, 7E, 7F and 8A). Minute lateral phasmids located short distance posterior to last pair of postanal papillae (Figs. 6C, 6D and 8A). Anterior cloacal lip with poorly developed unpaired median papilla. Tail conical, pointed, 87 long, without cuticular spines (Figs. 6C, 6D, 8A and 8B).

*Female*: Not known.

#### Remarks

This new species has no cuticular spines or protuberances on the male tail, as visible in Figure 11B (this being more apparent when further enlarged). Of the 12 species of *Raphidascaris* (*Ichthyascaris*) (see above), only *R*. *chirocentri*, *R*. *fasciati* n. sp., *R*. *fisheri*, *R*. *nemipteri*, *R*. *trichiuri*, and *R*. *vicentei* have the male tail tip smooth, without minute cuticular spines or protuberances. However, in contrast to *R*. *nudicauda* n. sp., *R*. *chirocentri* has longer spicules (315 μm vs. 201 μm), more numerous pairs of all caudal papillae (63 vs. 36) and those of postanal papillae (13 vs. 8) and a larger body of the male (body length 9.48 mm vs. 5.94 mm); *R*. *fasciati* n. sp. has much longer spicules (420–543 μm vs. 201 μm), more numerous pairs of caudal papillae (73–75 vs. 36) and those of postanal papillae (13 vs. 8) and the postanal papillae of the third pair from the posterior extremity are doubled (vs. single); *R*. *fischeri* has lateral margins of the lips having a small bulge posterior to the anterolateral sockets [5] (vs. such structures not visible); *R*. *nemipteri* possesses longer spicules (225–399 μm vs. 201 μm) and the postanal papillae of the third pair from the posterior extremity are doubled (vs. single); *R*. *trichiuri* has distinctly longer spicules (250–430 μm vs. 201 μm) and more numerous pairs of all caudal papillae (53 vs. 36) [16]; and *R*. *vincentei* has more numerous pairs of all caudal papillae (41–51 vs. 36) and those of postanal papillae (10–11 vs. 8) and more elongate lips without markedly protruding inner lobes [29]. *Raphidascaris nudicauda* n. sp. is the first representative of the subgenus *Ichthyascaris* reported from a fish belonging to the aulopiform family Synodontidae. Larval anisakids were reported from this fish [25, 27].

### *Raphidascaris* (*Ichthyascaris*) *euani* n. sp. Figures 9–11

urn:lsid:zoobank.org:act:8D0ED029-97A6-4A80-A7F2-ADAE05AAB294

**Figure 9:**
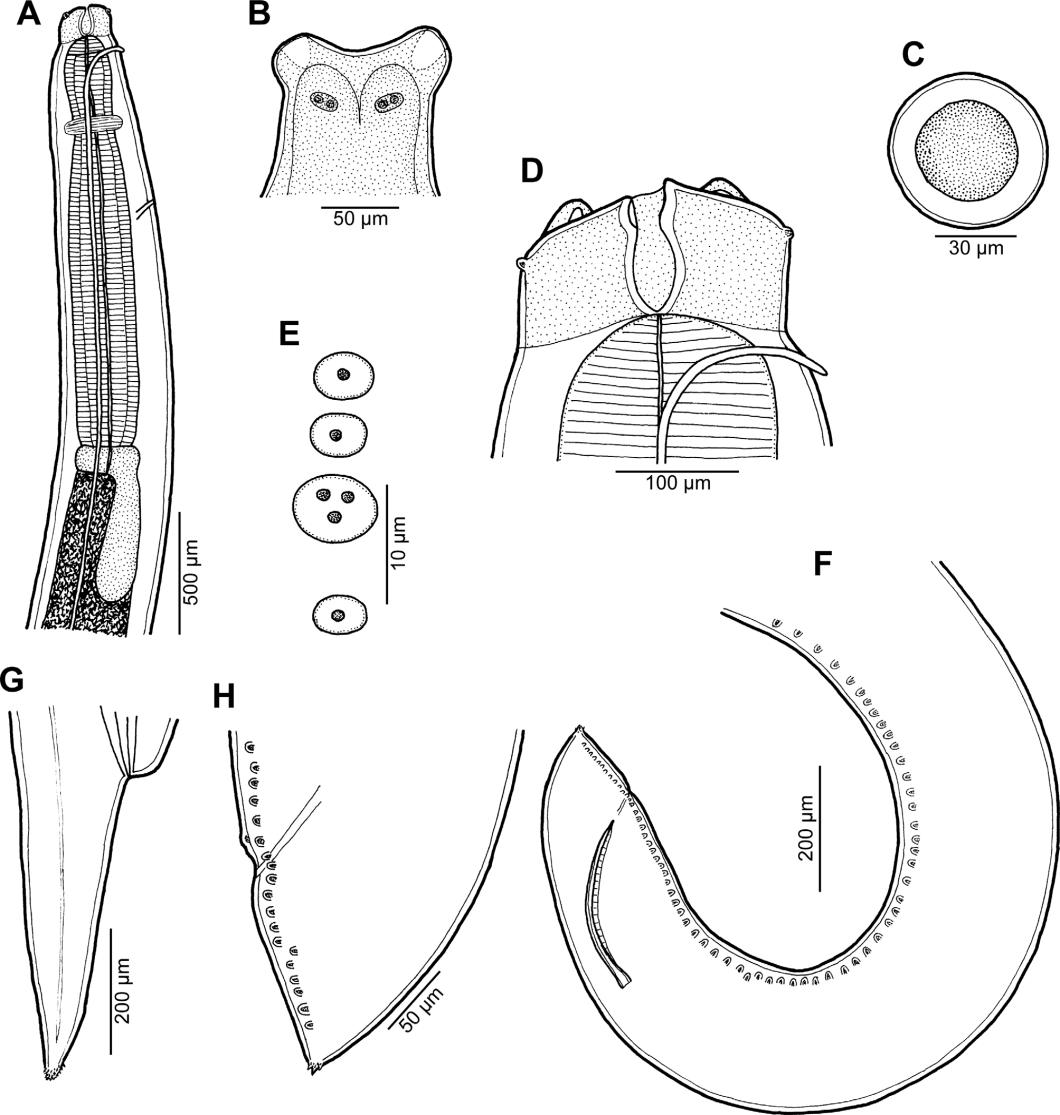
*Raphidascaris* (*Ichthyascaris*) *euani* n. sp. ex *Gymnocranius euanus*. (A) Anterior end of gravid female, lateral view; (B) dorsal lip; (C) egg; (D) cephalic end of female, lateral view; (E) postanal papillae of four posteriormost pairs on one side of body; (F) posterior end of male, lateral view; (G) tail of gravid female, lateral view; (H) caudal end of male, lateral view.

Type host: Japanese large-eye bream *Gymnocranius euanus* (Günther) (Lethrinidae, Perciformes).

Site of infection: Intestine.

Type locality: External slope of Récif Kué, off Nouméa, New Caledonia (collected 9 December 2008) (JNC2831).

Prevalence and intensity: 1 fish infected/60 fish examined (Justine et al. [11]); 14 nematodes.

Etymology: The specific name of this nematode relates to the genitive form of the species name of the type host.

Deposition of type specimens: Muséum National d’Histoire Naturelle, Paris, France (holotype, allotype and 7 paratypes, MNHN JNC2831) and Helminthological Collection, Institute of Parasitology, Biology Centre of the Czech Academy of Sciences, České Budějovice, Czech Republic (2 paratypes, N–1208).

#### Description

**Figure 10:**
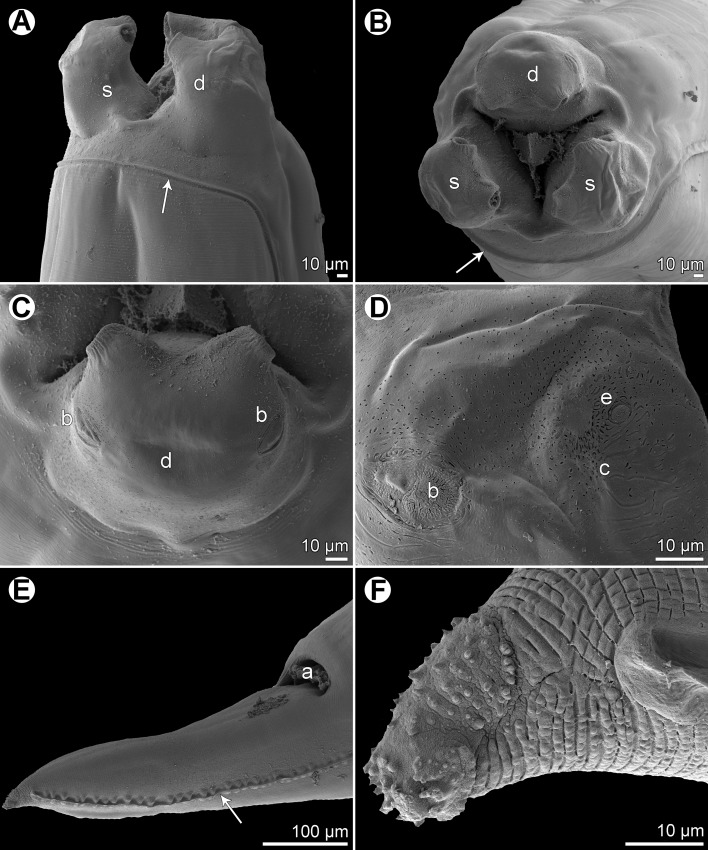
*Raphidascaris* (*Ichthyascaris*) *euani* n. sp., scanning electron micrographs of female. (A and B) Cephalic end, lateral and apical views, respectively (arrow indicates cuticular ala); (C) dorsal lip; (D) middle region of subventral lip; (E) tail, lateral view (arrow indicates lateral ala); (F) tail tip, lateral view. (a) Anus; (b) labial double papilla; (c) labial single papilla; (d) dorsal lip; (e) amphid; (s) subventral lip.

**Figure 11:**
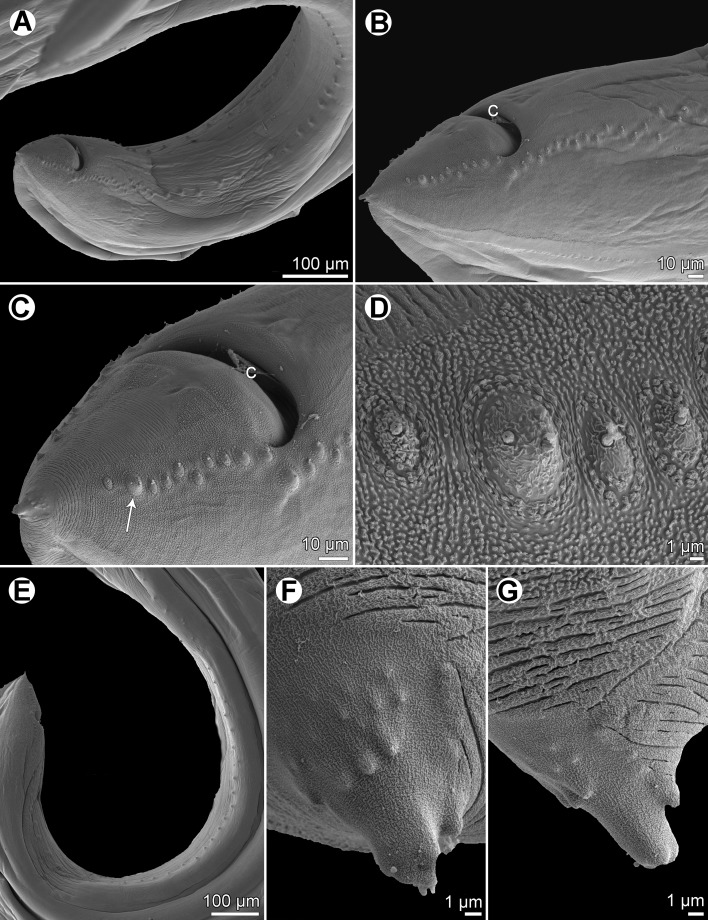
*Raphidascaris* (*Ichthyascaris*) *euani* n. sp., scanning electron micrographs of male. (A) Posterior end of body, subventral view; (B) posterior end (enlarged), ventrolateral view; (C) tail, ventrolateral view (arrow indicates postanal triple papilla); (D) postanal papillae of four posteriormost pairs (note distinct triple papilla); (E) posterior end of body (another specimen), lateral view; (F and G) tail tips of two different specimens. (c) Cloacal aperture.

*General*: Medium-sized nematodes with transversely striated cuticle (Figs. 10F and 11G). Lips nearly equal in size, without lateral membranous flanges; pulp with 2 distinct anterior lobes, each with terminal pocket-like depression (Figs. 9B, 9D and 10A–10D). Dorsal lip bears 2 subdorsal double papillae (Figs. 9B, 10B and 10C); each ventrolateral lip with 1 double subventral papilla, 1 small single papilla and amphid situated laterally (Fig. 10D). Interlabia absent. Narrow lateral alae extend along whole body length, united anteriorly close to ventrolateral lips on 1 side of body (Figs. 9A, 9D, 10A and 10B). Oesophagus short (Fig. 9A). Ventriculus transversely oval; ventricular appendix relatively short (Fig. 9A). Excretory pore well posterior to level of nerve ring (Fig. 9A). Tail of both sexes conical.

*Male* (4 specimens; measurements of holotype in parentheses): Body length 10.57–16.39 (16.39) mm, maximum width 313–530 (530). Lips 82–95 (95) long. Length of oesophagus 0.91–1.44 (1.36) mm, maximum width 136–272 (272), representing 8–10 (8)% of body length. Ventriculus 68–150 × 109–231 (150 × 231); ventricular appendix 381–734 (734) long, 68–136 (136) in maximum width. Posterior end curved ventrally (Figs. 9F, 11A and 11E). Spicules equal, alate, pointed, 318–396 (384) long, representing 2.34–3.75 (2.34)% of body length. Total of 60 (60) pairs of subventral papillae present, 48 (48) being preanals, 1 (1) pair adanals and 11 (11) pairs postanals; papillae of 24–25 (24) posteriormost preanal pairs and of postanal pairs very small; papillae of 1st postanal pair situated more laterally; postanal papillae of 2nd pair from posterior extremity triple (Figs. 9E, 9F, 9H and 11A–11D). Anterior cloacal lip with poorly developed unpaired median papilla. Phasmids not observed. Tail 135–147 (147) long, its tip provided with many cuticular spines or protuberances (Figs. 9H, 11F and 11G).

*Female* (1 compete [allotype] and 1 incomplete ovigerous specimens; measurements of allotype in parentheses, those of 4 nongravid specimens in brackets): Length of body 20.55 (20.55) mm and 18.36 mm in specimen with missing posterior end [12.60–17.08] mm, maximum width 680–721 (721) [462–585]. Lips 109–136 (109) [68–109] long. Length of oesophagus 1.81–1.84 (1.81) [1.17–1.39] mm, representing (9) [8–9]% of body length, maximum width 204–299 (204) [163–245]. Nerve ring and excretory pore 449–503 (449) [326–394] and 653–857 (653) [653–755], respectively, from anterior extremity. Ventriculus 136 × 204–245 (136 × 204) [95–109 × 150–204]; ventricular appendix 598–694 (694) [435–666] long, maximum width 109–167 (109) [54–109]. Vulva situated in anterior region of body, 4.19–4.26 (4.19) [2.45–3.33] mm from anterior extremity, at (20) [19–22]% of body length; vagina directed posteriorly from vulva. Eggs numerous, suboval to almost rounded, thin-walled, with uncleaved contents (Fig. 9C); size 42–60 × 30–54 (42–45 × 30–33) [–]. Tail (571) [408–449] long; tip with numerous minute cuticular spines distributed mainly on ventral side (Figs. 9G, 10E and 10F).

#### Remarks

In having the male tail tip covered with cuticular spines, *R*. (*I*.) *euani* n. sp. resembles the following eight species of the subgenus *Ichthyascaris*: *R*. *arii*, *R*. *fasciati* n. sp., *R*. *etelidis*, *R*. *gymnocraniae*, *R*. *longicauda*, *R*. *lophii*, *R*. *sillagoides*, and *R*. *spinicauda* n. sp. Of these, *R*. *fasciati* n. sp., *R*. *longicauda* and *R*. *lophii* differ from the new species in distinctly longer spicules (420–543 μm, 1.13–1.32 mm and 540–690 μm, respectively, vs. 318–396 μm), whereas *R*. *arii*, *R*. *gymnocraniae*, *R*. *sillagoides*, and *R*. *spinicauda* n. sp. have less numerous pairs of all caudal papillae (30–39, 33–38, 31–37, and 43, respectively, vs. 60) and those of postanal papillae (8, 8, 8–10, and 8, respectively, vs. 11). The total number of caudal papillae in *R*. *etelidis* is 60–65 (vs. 60) and that of postanal papillae 12–13 (vs. 11). However, postanal papillae of the second pair from the posterior extremity are triple in *R*. *euani* n. sp., this being unique among all *Raphidascaris* (*Ichthyascaris*) species, whereas the postanal papillae of the third (exceptionally fourth) pair from the posterior extremity in other species are double (*R*. *arii*, *R*. *fasciati*, *R*. *etelidis*) or single (*R*. *gymnocraniae*, *R*. *sillagoides*, *R*. *spinicauda*); no caudal papillae were described for *R*. *lophii*.

The only species of the subgenus *Ichthyascaris* reported from hosts belonging to the perciform family Lethrinidae is *R*. *gymnocraniae* described from *Gymnocranius audleyi* Ogilby (reported as *G*. *bitorquatus* Cockerell) (type host) and *Lethrinus miniatus* (Forster) (reported as *L. chrysostomus* Richardson) recorded from off the western Pacific coast of Australia (Queensland) [5]. Despite the fact that the present New Caledonian specimens were collected from the fish of the same genus (*Gymnocranius* Klunzinger) as the type host of *R*. *gymnocraniae* and from the nearby region, their morphology is very different (see above) and, consequently, they are considered to represent a new species.

*Gymnocranius euanus* has been thoroughly examined for parasites in New Caledonia, with 23 host-parasite combinations reported in 2010 [11]. Additional records since include digeneans [4], copepods [8] and larval anisakids [25]. The description of the present new species exemplifies again the high biodiversity of parasites in coral fish.

### *Raphidascaris* (*Ichthyascaris*) *elopsis* n. sp. Figures 12–14

urn:lsid:zoobank.org:act:FE09BB10-FB29-4A92-BDCB-095EB4A3F272

**Figure 12:**
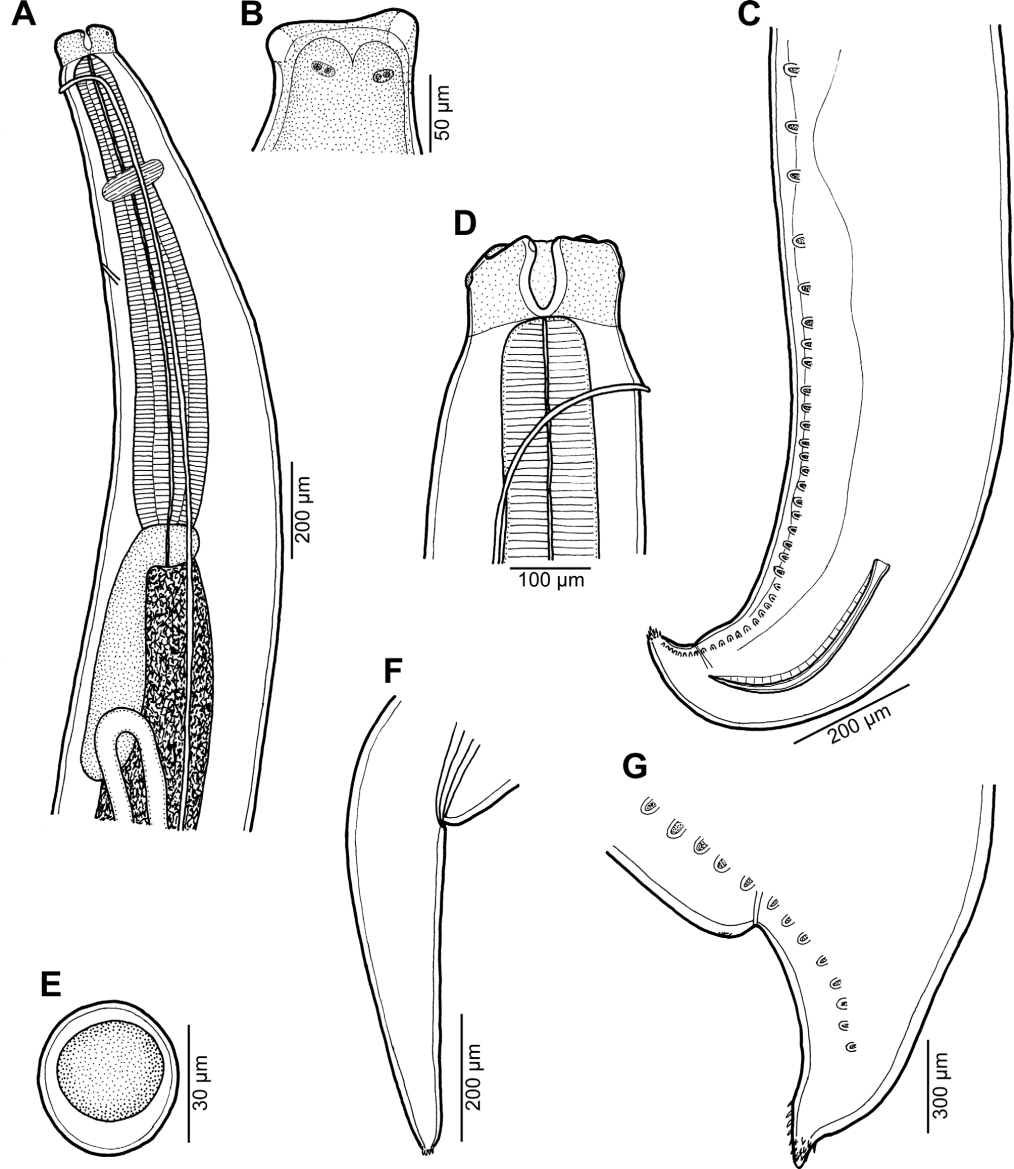
*Raphidascaris* (*Ichthyascaris*) *elopsis* n. sp. ex *Elops hawaiensis*. (A) Anterior end of male, lateral view; (B) dorsal lip; (C) posterior end of male, lateral view; (D) cephalic end of female, lateral view; (E) egg; (F) tail of female; (G) caudal end of male, lateral view.

Type host: Hawaiian ladyfish *Elops hawaiensis* Regan (Elopidae, Elopiformes).

Site of infection: Anterior portion of intestine.

Type locality: Côte Blanche, Nouméa, New Caledonia (collected 25 August 2003).

Prevalence, intensity and details about fish: 1 fish infected/1fish examined; *c*. 30 nematodes. The fish, JNC848, was 980 mm in fork length and 7000 g in weight.

Etymology: The specific name of this nematode relates to the genitive form of the generic name of the type host.

Deposition of type specimens: Muséum National d’Histoire Naturelle, Paris, France (holotype, allotype, and 39 paratypes, MNHN JNC848B) and Helminthological Collection, Institute of Parasitology, Biology Centre of the Czech Academy of Sciences, České Budějovice, Czech Republic (2 paratypes, N–1213).

#### Description

**Figure 13:**
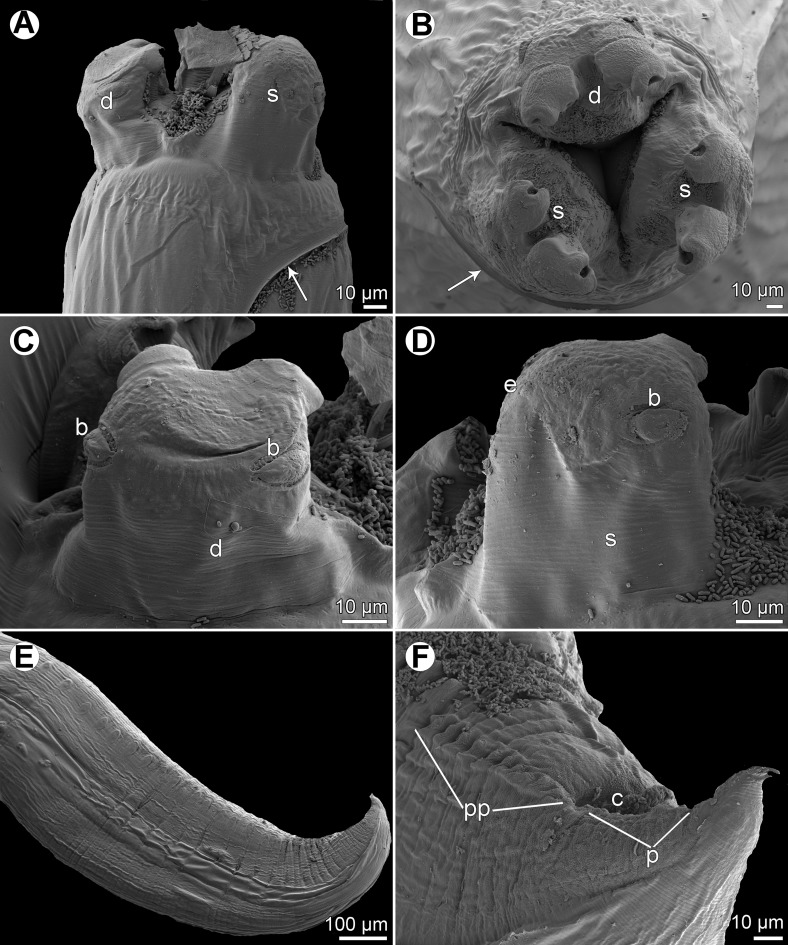
*Raphidascaris* (*Ichthyascaris*) *elopsis* n. sp., scanning electron micrographs of male. (A and B) Cephalic end, lateral and apical views, respectively (arrow indicates cuticular ala); (C) dorsal lip; (D) subventral lip; (E) posterior end of body, lateral view; (F) region of cloaca and tail, ventrolateral view. (b) Labial double papilla; (c) cloacal aperture; (d) dorsal lip; (e) amphid; (p) postanal papillae; (pp) posteriormost preanal papillae; (s) subventral lip.

**Figure 14:**
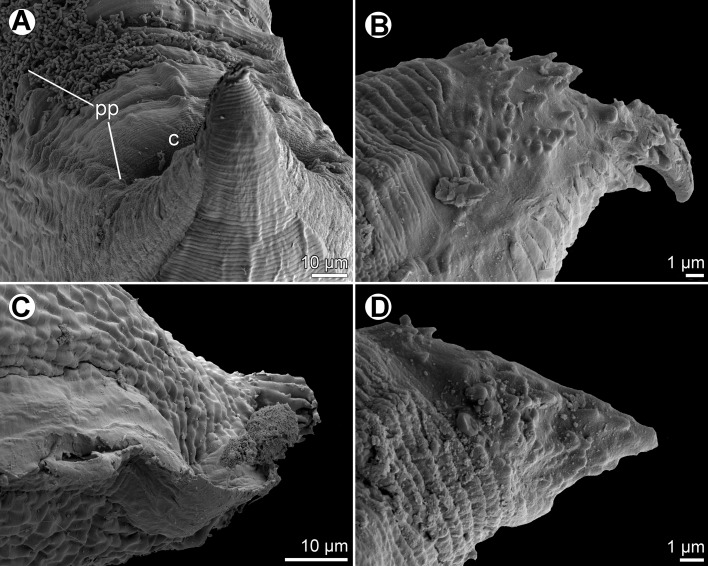
*Raphidascaris* (*Ichthyascaris*) *elopsis* n. sp., scanning electron micrographs. (A) Region of cloaca and tail, dorsoventral view; (B) tail tip of male, lateral view; (C) tail tip of gravid female, lateral view; (D) tail tip of male (another specimen), lateral view. (c) Cloacal aperture; (pp) preanal papillae of posteriormost pairs.

*General*: Medium-sized nematodes with transversely striated cuticle (Figs. 13E, 13F, 14A, 14B and 14D). Lips nearly equal in size, without lateral membranous flanges; pulp with 2 distinct anterior lobes, each with terminal pocket-like depression (Figs. 12B, 12D and 13A–13D). Dorsal lip bears 2 subdorsal double papillae (Figs. 12B, 13B and 13C); each ventrolateral lip with 1 double subventral papilla, 1 small single papilla and amphid situated laterally (Figs. 13B and 13D). Interlabia absent. Narrow lateral alae extend along whole body length, united anteriorly close to ventrolateral lips on 1 side of body (Figs. 12A, 12D, 13A and 13B). Oesophagus short (Fig. 12A). Ventriculus transversely oval; ventricular appendix relatively shortFig. 12A). Excretory pore well posterior to level of nerve ring (Fig. 12A). Tail of both sexes conical.

*Male* (5 specimens; measurements of holotype in parentheses): Body length 9.14–17.18 (17.18) mm, maximum width 367–653 (653). Lips 54–95 (95) long. Length of oesophagus 857–1360 (1360) mm, maximum width 122–245 (218), representing 8–9 (8)% of body length. Nerve ring and excretory pore 258–381 (367) and 462–762 (707), respectively, from anterior extremity. Ventriculus 68–136 × 95–204 (122 × 204); ventricular appendix 326–694 (694) long, 68–190 (150) in maximum width. Posterior end curved ventrally (Fig. 13E). Spicules equal, alate, pointed, 261–495 (426) long, representing 2.48–3.51 (2.48)% of body length. Total of 38–43 (43) pairs of subventral papillae present, 29–35 (35) being preanals and 8 (8) pairs postanals (Figs. 12C, 12G, 13F and 14A); papillae of 11–12 (12) posteriormost preanal pairs and of postanal pairs very small. Anterior cloacal lip with poorly developed unpaired median papilla. Phasmids not observed. Tail 72–129 (129) long, its tip provided with many cuticular spines or protuberances (Figs. 9C, 9G, 14B and 14D).

*Female* (4 ovigerous specimens; measurements of allotype in parentheses. Measurements of 1 nongravid specimen in brackets): Length of body 15.46–32.15 (32.15) [9.25] mm, maximum width 612–1319 (1319) [449]. Lips 68–136 (122) [54] long. Length of oesophagus 1.31–2.46 (2.07) [0.78] mm, representing 6–8 (6) [8]% of body length, maximum width 231–476 (476) [150]. Nerve ring and excretory pore 340–544 (530) [245] and 625–1,210 (925) [408], respectively, from anterior extremity. Ventriculus 109–204 × 204–354 (204 × 313) [82 × 136]; ventricular appendix 707–979 (775) [422] long, maximum width 95–299 (299) [95]. Vulva situated in anterior region of body, 3.26–4.62 (4.52) [1.59] mm from anterior extremity, at 14–21 (14) [17]% of body length; vagina directed posteriorly from vulva. Eggs numerous, suboval to almost rounded, thin-walled, with uncleaved contents (Fig. 12E); size 42–54 × 36–42 (48–51 × 42) [–]. Tail 354–598 (598) [340] long; tail tip truncated, with several minute cuticular outgrowths located on its top (Figs. 12F and 14C).

#### Remarks

Only seven species of *Raphidascaris* (*Ichthyascaris*), *R*. *arii*, *R*. *etelidis*, *R*. *euani* n. sp., *R*. *gymnocraniae*, *R*. *longispicula*, *R*. *sillagoides*, and *R*. *spinicauda* n. sp., have the tail tip of both males and females covered by many small cuticular spines or protuberances, as in the new species. Of them, *R*. *longispicula* differs by considerably longer spicules (1.13–1.32 mm), whereas the spicule lengths of the remaining six species are similar to those of *R*. *elopsis* n. sp. However, in contrast to the new species, the total number of pairs of caudal papillae is much higher in *R*. *etelidis* and *R*. *euani* (60–65 and 60, respectively, vs. 38–43); moreover, these two species also have more numerous pairs of postanal papillae (12–13 and 11, respectively, vs. 8). Whereas postanal papillae of the third pair from the posterior extremity are single in the new species as in *R*. *gymnocraniae*, *R*. *sillagoides* and *R*. *spinicauda*, these are double in *R*. *arii* and *R*. *etelidis*; postanal papillae of the same pair are single in *R*. *euani*, but those of the second pair from the posterior extremity are triple (vs. single).

Consequently, *R*. *elopsis* n. sp. cannot be differentiated from *R*. *gymnocraniae*, *R*. *sillagoides* and *R*. *spinicauda* based on the above-mentioned morphological features. However, the pairs of preanal papillae of *R*. *gymnocraniae* and *R*. *sillagoides* are less numerous (24–28 and 22–26, respectively, vs. 29–35), whereas those of postanal papillae may be more numerous (7–9 and 8–10, respectively, vs. 8); the vulva of gravid *R*. *sillagoides* females is located more posteriorly as compared to the new species (at 23–29% vs. 14–21% of the body length from the anterior extremity). The number of preanal papillae in *R*. *spinicauda* is identical to that in the new species (35 vs. 29–35), as well as that of postanal papillae (8 vs. 8), but both species differ from each other in the shape and structure of the female tail tip. Whereas the female tail tip of *R*. *spinicauda* is conical, sharply pointed, all covered with cuticular spines (Figs. 1C and 2D), that of *R*. *elopsis* n. sp. is truncated, with several outgrowths located on its top (Fig. 14C). Moreover, the hosts of these two species belong to different fish families and orders (Caesionidae, Perciformes *vs* Elopidae, Elopiformes).

This new species is the first representative of *Raphidascaris* (*Ichthyascaris*) described from a fish of the order Elopiformes.

### *Raphidascaris* (*Ichthyascaris*) *etelidis* Moravec et Justine, 2012

urn:lsid:zoobank.org:act:2BC62300-112C-42CC-B72A-321BA940CEAB

Hosts: Deep-water red snapper *Etelis carbunculus* Cuvier and deepwater longtail red snapper *E*. *coruscans* Valenciennes (both Lutjanidae, Perciformes).

Site of infection: Intestine.

Localities: For *E*. *carbunculus*, JNC2427, collected 28 November 2008: Off Passe de Dumbéa, off Nouméa, New Caledonia, 22°21′365 S, 166°14′041 E. For *E*. *coruscans*, JNC2448, collected 10 January 2008: Off Récif Kué, off Nouméa, New Caledonia, 22°35′186 S, 166°29′765 E.

Prevalence, intensity and details about fish: *E*. *carbunculus*: 1 fish infected/3 fish examined [12]; 7 nematodes. The fish examined, JNC2427, was 295 mm in fork length and 461 g in weight; a photograph has been deposited in Wikimedia (https://commons.wikimedia.org/wiki/File:Etelis_carbunculus_JNC2427_(Lutjanidae).JPG). *E*. *coruscans*: 1 fish infected/5 fish examined [12]; 2 nematodes. The fish, JNC2448, was 684 mm in fork length and 4200 g in weight.

Deposition of voucher specimens: Muséum National d’Histoire Naturelle, Paris, France, MNHN JNC2427 and JNC2448).

#### Remarks

This species has already been described in detail by Moravec and Justine [19] from lutjanid fishes *E*. *coruscans* (type host) and *Pristipomoides filamentosus* (Valenciennes) off New Caledonia. Whereas the present material from *E*. *carbunculus* contained both males and females, that from *E*. *coruscans* consisted only of a young female and one third-stage larva. The finding of this nematode species in *E*. *carbunculus* represents a new host record.

### *Raphidascaris* (*Ichthyascaris*) *sillagoides* (Bruce, 1990) Moravec et Nagasawa, 2002

Host: Silver sillago *Sillago sihama* (Forsskål) (Sillaginidae, Perciformes).

Site of infection: Intestine.

Locality: Fish market, Nouméa, New Caledonia (collected 12 October 2006).

Prevalence, intensity and details about fish: 1 fish infected/1 fish examined; 1 nematode. The fish, JNC2054, was 290 mm in fork length and 248 g in weight.

Deposition of voucher specimen: Muséum National d’Histoire Naturelle, Paris, France (MNHN JNC2054).

#### Description

*Nongravid female* (1 specimen): Small nematode; body length 14.88 mm, maximum width 544. Lips 95 long. Narrow lateral alae extend along almost whole body length, united anteriorly close to ventrolateral lips on one side of body. Oesophagus 1.46 mm long, representing 10% of body length, its maximum width 245. Nerve ring and excretory pore 435 and 816, respectively, from anterior end of body. Vulva 2.63 mm from anterior extremity, at 18% of body length. Uterus empty. Tail conical, 422 long, with posterior tip bearing numerous minute spines.

#### Remarks

The general morphology of the only available specimen is in agreement with the description of *R*. (*I*.) *sillagoides*, the only species of the subgenus *Ichthyascaris* parasitizing fishes of the family Sillagonidae [5]. The situation of the vulva in the present specimen (at 18% of the body length) as compared to that reported for *R*. *sillagoides* (23–29%) is apparently due to the fact that the New Caledonian specimen is represented by a small-sized, nongravid female, whereas the gravid females of *R*. *sillagoides* may be up to about 30 mm long. Taking into account that the present nematode was found in the congeneric host in the nearby region, it is considered to belong to *R*. *sillagoides*.

*Raphidascaris* (*I*.) *sillagoides* was described from *Sillago maculata* Quoy et Gaimard from off the western Pacific coast of Australia [5] and has not been recorded since. Accordingly, the present finding of this species from *S*. *sihama* in New Caledonia represents new host and geographical records.

### *Raphidascaris* (*Ichthyascaris*) sp. 1

Host: Trumpet emperor *Lethrinus miniatus* (Forster) (Lethrinidae, Perciformes).

Site of infection: Intestine.

Locality: External slope of Récif Kué, off Nouméa, New Caledonia (collected 9 December 2008).

Prevalence, intensity and details about fish: 1 fish infected/27 fish examined (Justine et al. [11]); 20 nematodes. The infected fish, JNC2824, was 430 mm in fork length and 1600 g in weight.

Deposition of voucher specimens: Muséum National d’Histoire Naturelle, Paris, France, MNHN JNC2824).

#### Remarks

Only a single juvenile male and numerous third- and fourth-stage larvae were collected from *L*. *miniatus*. Since no nominal species of *Raphidascaris* (*Ichthyascaris*) has so far been reported from fishes of the family Lethrinidae, it is highly probable that the present specimens belong to a new, undescribed species. In New Caledonia, *Raphidascaris* (*I*.) larvae (rarely also poorly preserved adults) were previously recorded from *Lethrinus genivittatus* Valenciennes, *L*. *miniatus* and *L*. *rubrioperculatus* Sato [19], which might belong to the same nematode species.

### *Raphidascaris* (*Ichthyascaris*) sp. 2

Host: White-spotted puffer *Arothron hispidus* (Linnaeus) (Tetraodontidae, Tetraodontiformes).

Site of infection: Intestine.

Locality: Near Ilôt Lebris, off Nouméa, New Caledonia, 21°49′622 S, 166°45′353 E (collected 25 October 2007).

Prevalence, intensity and details about fish: 1 fish infected/2 fish examined; 3 nematodes. The infected fish, JNC2335, was 394 mm in fork length and 1369 g in weight.

Deposition of voucher specimens: Muséum National d’Histoire Naturelle, Paris, France, MNHN JNC2335).

#### Description

*Female* (1 gravid specimen): Length of body 38.08 mm, maximum width 802. Lips 136 long. Length of oesophagus 2.33 mm, representing 6% of body length, maximum width 354. Nerve ring and excretory pore 530 and 544, respectively, from anterior extremity. Ventriculus 177 × 299; ventricular appendix 870 long, maximum width 163. Vulva situated in anterior region of body, 4.62 mm from anterior extremity, at 12% of body length; vagina directed posteriorly from vulva. Eggs numerous, suboval to almost rounded, thin-walled, with uncleaved contents; size 54–57 × 45–51. Tail conical, 625 long; tail tip with numerous minute cuticular spines.

#### Remarks

One gravid female and two body fragments of small larvae were collected from the digestive tract of *A*. *hispidus*. Considering the host specificity of *Raphidascaris* (*Ichthyascaris*) species at the level of host fish families and the fact that previously no representatives of this anisakid subgenus were recorded from tetraodontiform fishes, apparently the present specimens belong to an undescribed species. The available female is the longest one among all known species of *Ichthyascaris*, only the female of *R*. (*I*.) *trichiuri* may nearly reach the same length (up to 37.8 mm), but its tail tip lacks minute cuticular spines. Nevertheless, in having no males, we refrain from establishing a new species for these nematodes.

### *Hysterothylacium alatum* Moravec et Justine, 2015

urn:lsid:zoobank.org:act:DDAC2328-8247-4D87-B38B-68990BA2CDA9

Host: Leopard coralgrouper *Plectropomus leopardus* (Lacepède) (Serranidae, Perciformes).

Site of infection: Stomach.

Locality: Near Ilôt Sainte Marie, off Nouméa, New Caledonia (collected 4 April 2003).

Prevalence, intensity and details about fish: 1 fish infected/24 fish examined [10]; 1 nematode. The infected fish specimen, JNC381, was 285 mm in total length and 301 g in weight.

Deposition of voucher specimen: Muséum National d’Histoire Naturelle, Paris (MNHN JNC381A).

#### Description

*Female* (1 gravid specimen): Body elongate, yellowish in colour, 44.23 mm long, maximum width 558. Cuticle transversely striated. Broad cervical alae *c*. 1.36 mm long and 109 in maximum width. Lips 87 long; length of interlabia 27. Length of oesophagus 2.18 mm, representing 3% of body length; maximum width 163. Nerve ring and excretory pore 612 and 612, respectively, from anterior extremity. Ventriculus 109 × 122; ventricular appendix 1.29 mm long, maximum width 68. Intestinal caecum 367 long, maximum width 95. Caecum to ventricular appendix length ratio 1:35. Vulva postequatorial, 23.56 mm from anterior end of body, at 53% of body length; vulval lips not protruding. Vagina directed posteriorly from vulva, 884 long and 54 wide. Several somewhat narrowed eggs present only in vagina, size 60–69 × 36–48. Tail conical, slender, 585 long; tip rounded, bearing numerous small cuticular protuberances.

#### Remarks

The only available specimen (young gravid female) is morphologically identical to those of *H*. *alatum*, described from the congeneric host *Plectropomus laevis* (Lacepède) off New Caledonia [21], and, consequently, it is considered to belong to this species. The present finding of *H*. *alatum* in *P*. *leopardus* represents a new host record for this nematode species.

### *Hysterothylacium epinepheli* (Yamaguti, 1941) Deardorff et Overstreet, 1981 Figures 15–17

Host: Highfin grouper *Epinephelus maculatus* (Bloch) (Serranidae, Perciformes).

**Figure 15:**
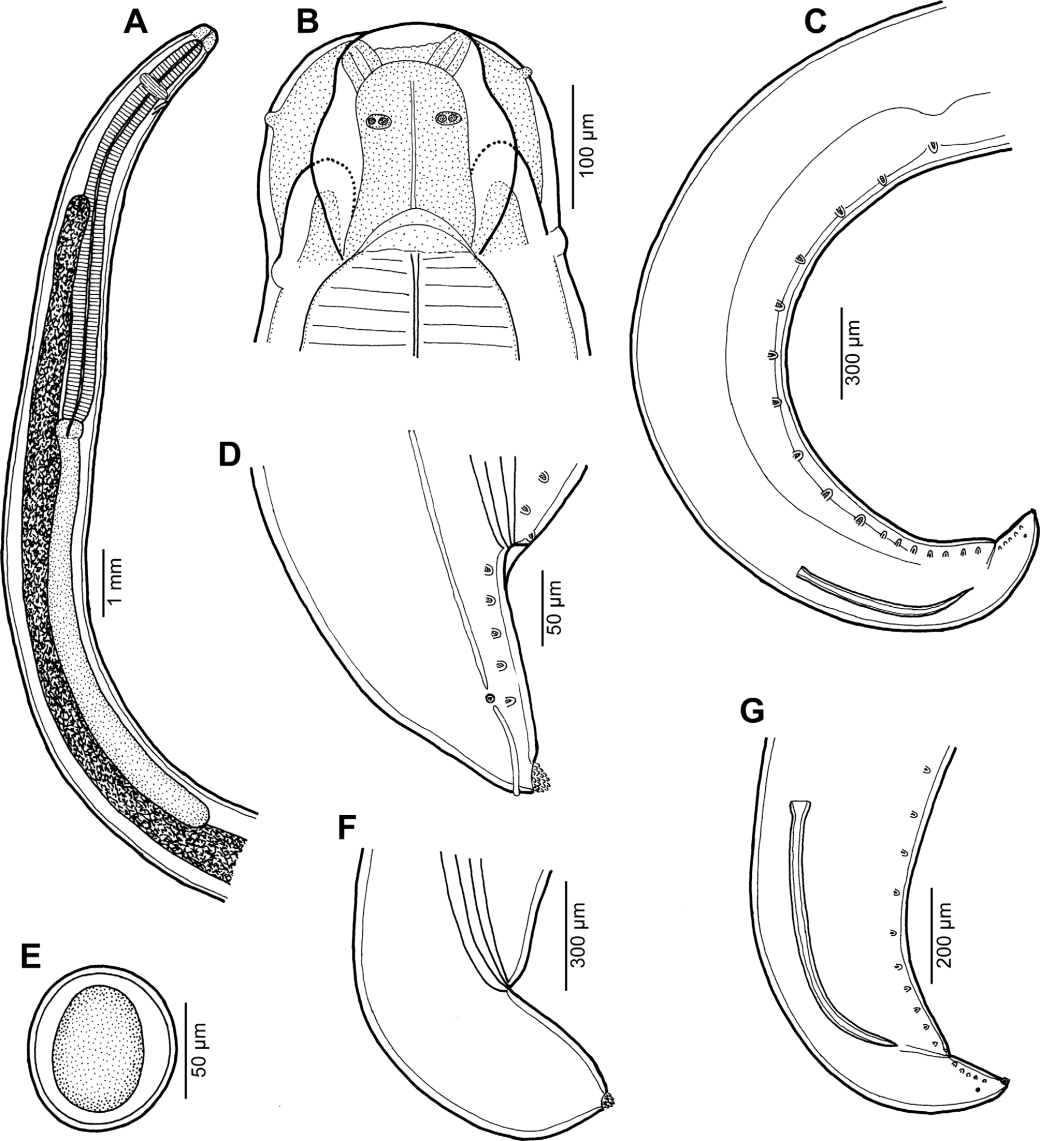
*Hysterothylacium epinepheli* (Yamaguti, 1941) ex *Epinephelus maculatus*. (A) Anterior end of male, lateral view; (B) cephalic end of male, dorsal view; (C) posterior end of male, lateral view; (D) tail of male, lateral view; (E) egg; (F) tail of gravid female, lateral view; (G) caudal end of male, lateral view.

Site of infection: Intestine.

Locality: Récif Toombo, off Nouméa, New Caledonia, 22°32′263 S, 166°27′267 E (collected 13 December 2005).

Prevalence, intensity and details about fish: 1 fish infected/38 fish examined [10]; 3 nematodes. The infected fish specimen, JNC1682, was 500 mm in total length and 1500 g in weight.

Deposition of voucher specimens: Helminthological Collection, Institute of Parasitology, Biology Centre of the Czech Academy of Sciences, České Budějovice, Czech Republic (male and female body ends mounted on SEM stub) (Cat. No. N–1212). Muséum National d’Histoire Naturelle, Paris (2 males and 1 female, both without body ends, in 70% ethanol) (MNHN JNC1682).

#### Description

**Figure 16:**
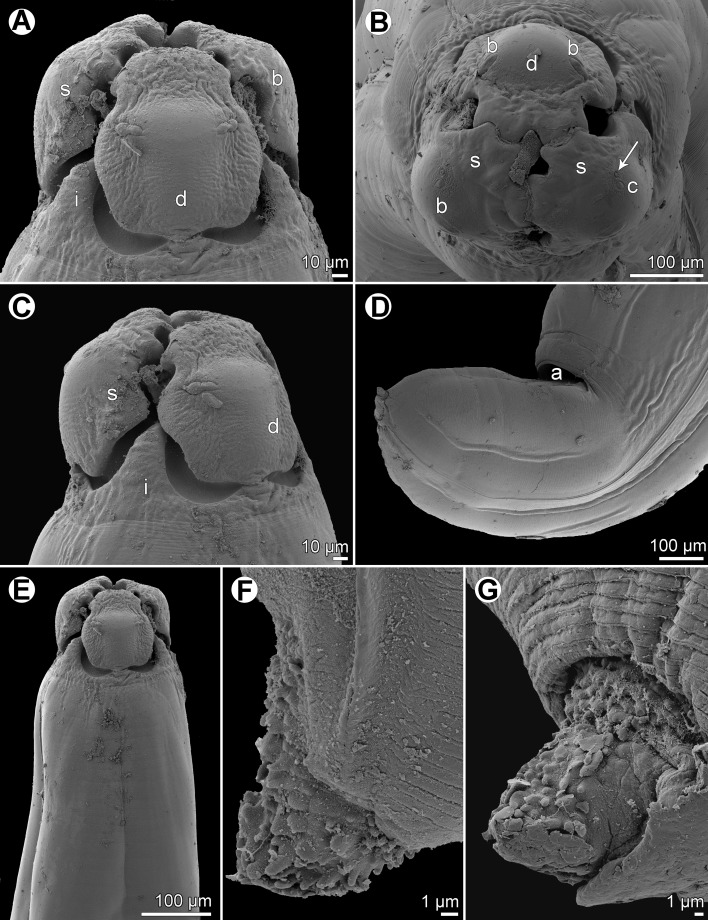
*Hysterothylacium epinepheli* (Yamaguti, 1941), scanning electron micrographs. (A) Cephalic end, dorsal view; (B and C) cephalic end, apical and lateral views, respectively (arrow indicates amphid); (D) female tail, lateral view; (E) anterior end of body, dorsal view; (F) tail tip of male, lateral view; (G) tail tip of female, lateral view. (a) Anus; (b) labial double papilla; (c) labial single papilla; (d) dorsal lip; (i) interlabium; (s) subventral lip.

**Figure 17:**
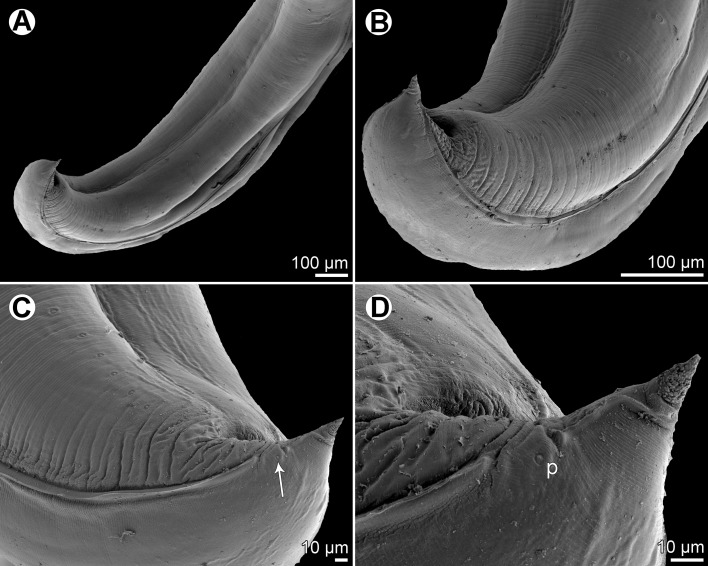
*Hysterothylacium epinepheli* (Yamaguti, 1941), scanning electron micrographs of male. (A) Posterior end of body, ventrolateral view; (B) enlarged posterior end, ventrolateral view; (C) same from opposite side (arrow indicates phasmid); (D) tail, lateral view. (p) phasmid.

*General*: Body large, elongate, yellowish in colour, with transversely striated cuticle (Figs. 16F, 16G, 17B and 17C). Maximum width near middle of body. Lips almost equal in size (dorsal lip slightly smaller than ventrolateral lips), somewhat longer than wide, with narrow bases; their lateral flanges widest at middle of lips; pulp with 2 anteriorly protruding lobes. Dorsal lip with 2 subdorsal double papillae; each subventral lip with 1 double subventral papilla, 1 small single papilla and amphid situated laterally (Figs. 15B, 16A–16C and 16E). Interlabia well developed, nearly 1/2 length of lips (Figs. 15B, 16A and 16C). Lateral alae very narrow, extending posteriorly almost to end of tail (Figs. 15D, 16F and 17B–17D). Deirids not observed. Oesophagus almost cylindrical, long. Nerve ring encircles oesophagus at about one fifth of its length. Ventriculus small, almost spherical; ventricular appendix long, narrow. Intestinal caecum distinctly shorter than ventricular appendix (Fig. 15A); caecum to ventricular appendix length ratio 1:1.9–2.6. Excretory pore just posterior to level of nerve ring (Fig. 15A). Tail of both sexes conical; tip with numerous minute cuticular outgrowths difficult to observe under LM.

*Male* (2 specimens): Length of body 26.59–31.92 mm, maximum width 748–898. Lips 163–190 long; length of interlabia 68–82. Length of oesophagus 3.20–3.35 mm, representing 10–13% of body length; maximum width 231–326. Nerve ring and excretory pore 639–748 and 666–748, respectively, from anterior extremity. Ventriculus 177–204 × 258–272; ventricular appendix 3.58–4.60 mm long, maximum width 245–340. Intestinal caecum 1.74–1.93 mm long, maximum width 218–231. Caecum to ventricular appendix length ratio 1:1.9–2.6; caecum represents 38–58% of entire oesophagus length. Posterior end of body curved ventrally. Spicules equal, 653–720 long, representing 2.3–2.5% of body length (Figs. 15C and 15G). Total of 24 pairs of poorly visible subventral papillae present, 17 being preanals and 7 postanals; papillae of 7 posteriormost preanal and postanal pairs very small (Figs. 15C, 15D, 15G and 17B–17D). One papilla-like ventromedian organ located on anterior cloacal lip present (Figs. 15D, 17C and 17D). Pair of small lateral phasmids situated between 2 last pairs of postanal papillae (Figs. 15D, 17C and 17D). Tail conical, 122–163 long; tail tip bearing numerous minute protuberances (Figs. 15D, 16F, 17C and 17D).

*Female* (1 ovigerous specimen): Length of body 59.23 mm, maximum width 1.63 mm. Lips 258 long; length of interlabia 109. Length of oesophagus 4.90 mm, representing 8% of body length; maximum width 340. Nerve ring and excretory pore 952 and 979, respectively, from anterior extremity. Ventriculus 272 × 272; ventricular appendix 5.92 mm long, maximum width 313. Intestinal caecum 2.72 mm long, maximum width 367. Caecum to ventricular appendix length ratio 1:2.2; caecum represents 46% of entire oesophagus length. Vulva preequatorial, 18.77 mm from anterior end of body, at 32% of body length; vulval lips not elevated. Vagina directed posteriorly from vulva. Uterus containing numerous, almost spherical eggs 68–82 in diameter (Fig. 12E). Tail conical, broad, 558 long (Figs. 12F and 16D); tip rounded, bearing numerous minute cuticular outgrowths (Fig. 16G).

#### Remarks

The morphology and measurements of the present nematodes are almost identical to those of *Hysterothylacium epinepheli*, a species described by Yamaguti [32] from the congeneric host *Epinephelus akaara* (Temminck et Schlegel) off Japan, and, therefore, the New Caledonian specimens are considered to belong to this species. The original description of *H*. *epinepheli*, based on LM, is rather incomplete. Therefore, the present redescription of this species, based on both LM and SEM examinations, provides some morphological features (e.g. the presence of minute outgrowths on the tail tip, lateral alae, phasmids, excretory pore, the size of eggs), which were overlooked or given inaccurately by Yamaguti [32]. It should be noted that, in this species, the male caudal papillae and the minute outgrowths on the tail tip of both sexes are very difficult to observe under LM.

Yamaguti [32] collected *H*. *epinepheli* from *E*. *akaara* in the Seto Inland Sea, Japan and this nematode species has not been recorded since. Consequently, the present finding of *H*. *epinepheli* in *E*. *maculatus* in New Caledonian waters represents new host and geographical records.

*Epinephelus maculatus* has been thoroughly examined for parasites in New Caledonia, with 36 host-parasite combinations reported in 2010 [10]. Additional records since include digeneans [3], trypanorhynch cestodes [2], and larval anisakids [25]. The present record of *H*. *epinepheli* exemplifies again the high biodiversity of parasites in coral fish.

## Discussion

According to Li et al. [15], molecular phylogenetic analyses divide the superfamily Ascaridoidea Railliet et Henry, 1915 into six main monophyletic clades representing the families, including Anisakidae and Raphidascarididae Hartwich, 1954. However, in our opinion, it is premature to create a new classification system of ascaridoids mostly based on molecular data, because only a very small number of these species have been examined by molecular methods and representatives of many ascaridoid genera have not been examined at all. Therefore, for the time being, we prefer to use for higher taxa the system of the widely used Keys to the nematode parasites of vertebrates [1], principally based on nematode morphology, where Raphidascaridinae is considered a subfamily of the Anisakidae.

As mentioned above, only six nominal species of adult anisakids were previously reported as parasites of marine fishes in New Caledonian waters. Therefore, the present data, including descriptions of five new species of *Raphidascaris* (*Ichthyascaris*), two other specifically unidentified congeneric species and two previously unreported *Hysterothylacium* and *Raphidascaris* (*Ichthyascaris*) species from off New Caledonia, extend considerably the knowledge of the fauna of these parasites in this region. In particular, the recorded species diversity within *Raphidascaris* (*Ichthyascaris*) is remarkable, with eight described and possibly an additional three undescribed New Caledonian species, exhibiting their host specificity at least at the level of fish families. However, it is necessary to note that some taxonomically important morphological features of these nematodes are not easily visible under LM and need to be studied by SEM.
